# Pneumoperitoneum

**Published:** 1915-03

**Authors:** 


**Pneumoperitoneum.** J. D. Malcolm, "Lancet," January 9, 1915, reports a case developing three days after resection of a carcinomatous rectum. He thinks it impossible that gas could either have escaped through a macroscopic opening without infection of the peritoneum or that it could have permeated through the wall of the bowel and ascribes the development of gas to some non-pyogenic bacterium, stating that only two are known which produce gas without putrefaction: B. aerogenes capsulatus of Welch and B. oedematis aerobius of San Felice.

